# The Changing Epidemiology of Viral Hepatitis in a Post-Soviet Country—The Case of Kyrgyzstan

**DOI:** 10.3390/pathogens12080989

**Published:** 2023-07-28

**Authors:** Manas K. Akmatov, Nurgul J. Beisheeva, Asylbek Z. Nurmatov, Sattarova J. Gulsunai, Kylychbekova N. Saikal, Aisuluu A. Derkenbaeva, Zamira O. Abdrahmanova, Jana Prokein, Norman Klopp, Thomas Illig, Omor T. Kasymov, Zuridin S. Nurmatov, Frank Pessler

**Affiliations:** 1 TWINCORE, Centre for Experimental and Clinical Infection Research, 30625 Hannover, Germany; makmatov@zi.de; 2 Currently at Central Research Institute of Ambulatory Health Care, 10587 Berlin, Germany; 3 National Scientific and Practical Center for Control of Viral Infections, Bishkek 720005, Kyrgyzstan; b_nurgul77@mail.ru (N.J.B.); asilbeknurmat@mail.ru (A.Z.N.); s.gulsun87@mail.ru (S.J.G.); skylychbekova15@gmail.com (K.N.S.); aysuluu88@list.ru (A.A.D.); abdrahmanova.z@list.ru (Z.O.A.); zuridin@mail.ru (Z.S.N.); 4 Hannover Unified Biobank, Hannover Medical School, 30625 Hannover, Germany; prokein.jana@mh-hannover.de (J.P.); klopp.norman@mh-hannover.de (N.K.); illig.thomas@mh-hannover.de (T.I.); 5 Scientific and Production Centre for Preventive Medicine, Ministry of Health, Bishkek 720005, Kyrgyzstan; logvinehko@mail.ru; 6 Helmholtz Centre for Infection Research, 38124 Braunschweig, Germany

**Keywords:** endemicity pattern, hepatitis A, hepatitis B, hepatitis C, hepatitis D, hepatitis E, seroprevalence, general population, Kyrgyzstan, post-Soviet country, WHO European Region

## Abstract

Historically, viral hepatitis has been a considerable public health problem in Central Asian countries, which may have worsened after the dissolution of the Soviet Union. However, up-to-date seroepidemiological studies are lacking. The aim of the present study was, therefore, to provide current estimates of the seroprevalence of viral hepatitis in Kyrgyzstan, one of the economically least developed countries in the region. We conducted a population-based cross-sectional study in 2018 in the capital of Kyrgyzstan, Bishkek (*n* = 1075). Participants, children and adults, were recruited from an outpatient clinic. The data were collected during face-to-face interviews. A blood sample (6 mL) was collected from each participant and tested with ELISA for the presence of serological markers for five viral hepatitides (A, B, C, D, and E). Post-stratification weighing was performed to obtain nationally representative findings. The overwhelming majority of the study participants were positive for anti-HAV (estimated seroprevalence, 75.3%; 95% confidence interval, 72.5–77.9%). The weighted seroprevalence estimates of HBsAg, anti-HCV, and anti-HDV were 2.2% (1.5–3.3%), 3.8% (2.8–5.1%), and 0.40% (0.15–1.01%), respectively. Anti-HEV seropositivity was 3.3% (2.4–4.5%). Of the 33 HBsAg-positive participants, five (15%) were anti-HDV-positive. Our study confirms that Kyrgyzstan remains a highly endemic country for hepatitis virus A and C infections. However, seroprevalences of HBV and HDV were lower than previously reported, and based on these data, the country could potentially be reclassified from high to (lower) intermediate endemicity. The observed anti-HEV seroprevalence resembles the low endemicity pattern characteristic of high-income countries.

## 1. Introduction

Viral hepatitis is considered a substantial public health issue in the former Soviet republics of Central Asia [1]. However, there are surprisingly few robust data points about its current epidemiology in these countries. Kyrgyzstan, one of the poorest countries in the region, traditionally belongs to the high-endemic countries for viral hepatitis [2]. During 2000–2008, the most prevalent viral hepatitis in Kyrgyzstan was hepatitis A (HAV); of all reported hepatitides, A was the most prevalent (61%), followed by B (17%), C (4.5%), and D (1.7%) [3]. Jacobsen et al. classified Kyrgyzstan as a country with an intermediate prevalence of HAV, with more than 50% of the population infected with the virus by the age of 15 [4]. The World Health Organization (WHO) classified Kyrgyzstan as a country with high endemicity regarding HBV, which was confirmed in the last systematic review of global HBV seroprevalence [5]; an estimated 550,000 Kyrgyzsians were positive for HBV, corresponding to a seroprevalence of 10.3% (95% confidence intervals, CI: 8.6–12.4%). Of note, Kyrgyzstan was the only high endemic country (i.e., HBsAg prevalence of ≥8%) out of the 44 countries of the WHO European Region [5]. However, this estimate was based on a single small-scale study from 1992 [6]. More recently, we estimated the HBsAg seroprevalence in a large sample of blood donors from 2013 to 2015 (*n* = 37,165) [7]; the estimate was almost three-times lower than the above-mentioned estimate from 1992 [6] (3.6% [95% CI: 3.4–3.8%] vs. 10.3% [8.6–12.4%]). In addition, there was a decreasing trend in HBV seropositivity during this time period. Regarding HCV, Kyrgyzstan was classified as a high-endemic country with a prevalence of >3.5% [8]. Recently, Boteju et al. estimated the pooled HCV prevalence in the general Kyrgyz population to be 2.0% (95% CI: 1.7–2.4%) [9]. A somewhat higher seroprevalence of 3.1% (95% CI: 3.0–3.3%) was observed among blood donors in the above-mentioned study [7]. Not much is known about hepatitis D and E. Outbreaks of hepatitis E in Kyrgyzstan were reported in the 1990s [10,11], but there are no data about its seroprevalence either during that time period or later [12]. Thus, there is a clear need for current data on the seroprevalences of the currently known viral hepatitides in Kyrgyzstan. The aim of the present study was, thus, to fill this data gap by way of a cross-sectional study in the general Kyrgyz population, including equal proportions of female and male participants and spanning all age groups from 1 through 72 years.

## 2. Materials and Methods

### 2.1. Study Design and Data

We conducted a population-based cross-sectional study in the capital of Kyrgyzstan, Bishkek, in the year 2018. Kyrgyzstan is one of the five former Soviet republics located in Central Asia and had 6,256,730 inhabitants at that time. Approximately one-sixth of the total population lives in Bishkek. The primary health care providers in Bishkek are family medicine centers (FMC), which represent the largest outpatient clinics (formerly known as polyclinics). In total, 19 FMCs are responsible for outpatient health care in Bishkek and adjacent urban areas. Potential study participants were recruited from FMC no. 11, which covers a population of approx. 30,000 inhabitants in Bishkek. Inhabitants with permanent residence in Bishkek are registered in FMCs. Each FMC possesses a list of registered inhabitants, including their addresses and contact information. The study team consisted of one physician and two study nurses. Potential study participants (or parents) were selected randomly and contacted by the study team by phone to participate in the study. The study aimed to recruit 240 children and adolescents (≤18 years old) and 760 adults (>18 years old). However, the recruitment of children, in particular those younger than 10 years, turned out to be difficult. The final sample comprised 207 children and adolescents (≤18 years) and 868 adults.

### 2.2. Questionnaire

Sociodemographic and medical data were collected during face-to-face interviews. The 49-item questionnaire collected information about sociodemographic data (sex, age, ethnicity, marital status, education level, and place of birth); self-perceived health status; infectious diseases (upper and lower respiratory tract infections, gastrointestinal infections, labial herpes, skin and mucosal infections, and bladder and kidney infections) [13,14]; noncommunicable diseases (e.g., diabetes, myocardial infarction, asthma, rheumatoid arthritis, and cancer); contacts with patients with known hepatitis B, C, or D infection; medical procedures (e.g., intravenous injections, organ transplantation, transfusion of blood products, operations, and tooth extraction); cosmetic procedures (e.g., tattoo, manicure, and pedicure); pharmacotherapy (e.g., antibiotics, corticosteroids); and illicit drug use.

### 2.3. Laboratory Analysis

A blood sample (6 mL) was drawn from each participant. Enzyme-linked immunoassays (ELISA) were performed using a microplate spectrophotometer (ELx800, BioTek, Winooski, VT, USA). The ELISA kits Vektogep A—IgG (Vector-Best, Novosibirsk, Russia) were used to test for the presence of antibodies against HAV (anti-HAV). The kits DS-EIA-HBsAg, EIA-anti-HCV, EIA-anti-HDV, and DS-EIA-anti-HEV-G (RPC Diagnostic Systems, Nizhny Novgorod, Russia) were used to test for the presence of HBsAg and for antibodies against HCV (anti-HCV; HCV core antigen, NS3Ag, NS4Ag, and NS5-Ag), HDV (anti-HDV), and HEV (anti-HEV), respectively. All positive samples were retested once using the same ELISA assays and the same blood sample. Samples were considered positive if the results were positive in both tests. All ELISA kits used in this study are licensed for use by the Ministry of Health in Kyrgyzstan.

### 2.4. Notification Data

We used publicly available national notification data for viral hepatitis in Kyrgyzstan [15]. In brief, a viral hepatitis surveillance system that captures acute viral hepatitis has existed since Soviet times. Notification of chronic viral hepatitis was introduced in 2010. Etiological differentiation was only possible for a small proportion of notified cases.

### 2.5. Statistical Analysis

Initially, we calculated the crude seroprevalence of each viral hepatitis. Since some population subgroups were under- or oversampled, we applied post-stratification weights using sex and 10-year age groups to obtain nationally representative seroprevalence estimates. The sex and age distribution of the general Kyrgyz population was obtained from the National Statistical Committee of the Kyrgyz Republic (www.stat.kg, accessed on 5 February 2023). Furthermore, we estimated sex-, age-, and ethnicity-adjusted seropositivity using logistic regression analysis. Four separate models were created (HAV, HBV, HCV, and HEV). A model for HDV was not created because there were only six individuals with positive serology. Analyses were performed with IBM SPSS Statistics for Windows, version 19 (IBM Corporation, Armonk, NY, USA).

## 3. Results

### 3.1. Demographic and Health-Related Description of the Study Population

Descriptive characteristics of the study population are presented in Table 1. Briefly, there was a slightly lower proportion of males than females (46% vs. 54%). Around 20% of the participants were children and adolescents. The overwhelming majority of the study population was of Kyrgyz origin (79%), followed by Russians (12%). The proportions of study participants with diabetes, myocardial infarction, and asthma were 1.3%, 0.84%, and 0.74%, respectively (Table 1). Every sixth participant reported a poor/fair health status.

### 3.2. Seroprevalence of Viral Hepatitis

The crude and weighted seroprevalences of the five forms of viral hepatitis are presented in Table 2. The overwhelming majority of the study participants were positive for anti-HAV (84%). The crude prevalences of anti-HCV, anti-HEV, and HBsAg were 4.7, 4.4, and 3.1%, respectively. Of the 33 HBsAg-seropositive participants, 30 (91%) were anti-HBc-positive, indicating acute or chronic infection. Six participants (0.60%) were seropositive for anti-HDV. Of all 33 HBsAg-positive participants, five (15%) were also anti-HDV-positive. One anti-HDV positive participant was anti-HBc positive but HBsAg negative, which may point to a resolved infection or to a “low grade” chronic infection. The seroprevalence decreased for all viral hepatitides after poststratification weighting (Table 2, sixth column).

### 3.3. Seroprevalence by Sex, Age, and Ethnicity

**Figure 1:**
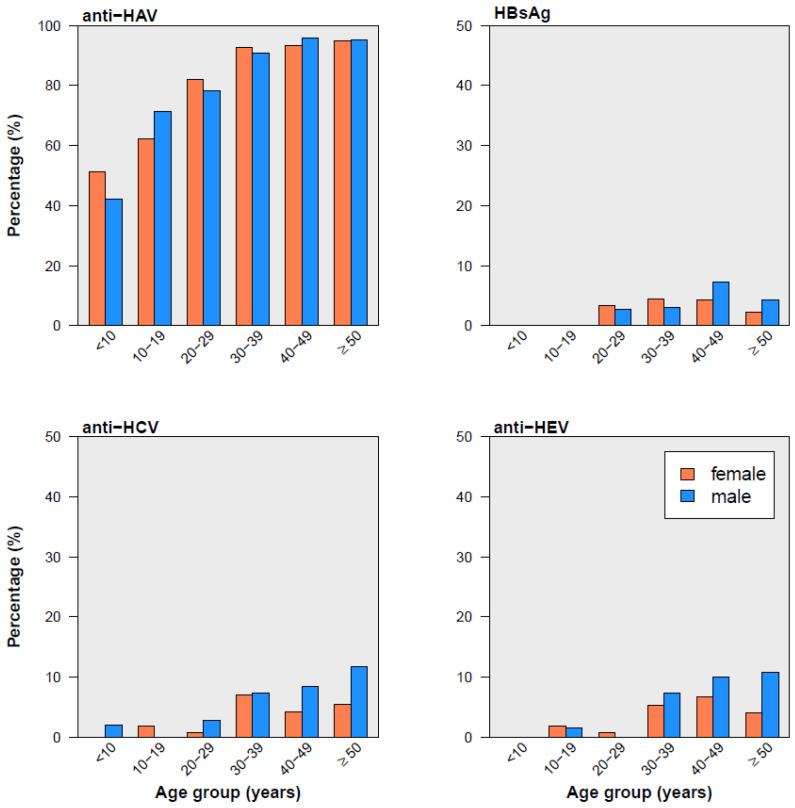
Seroprevalence of four viral hepatitides by sex and age groups. Anti-HAV, anti-HCV, and anti-HEV are antibodies against HAV, HCV, and HEV, respectively; HBsAg is hepatitis B surface antigen. The sex- and age-distribution of anti-HDV positive participants is not presented due to the low number (*n* = 6).

Seroprevalence increased with advancing age for all viral hepatitides except HDV (Figure 1 and Table 3). This increase was the highest for HAV, as the risk of seropositivity increased by 8% per additional year of the participant’s age. By age 30, the proportion of HAV seropositives had reached almost 90% and increased slightly afterwards (Figure 1).

The six study participants with HDV-seropositive results (4 males, 2 females) were between 33 and 58 years old. In terms of sex-related differences, HCV constituted a notable exception in that the risk of HCV positivity was about two-times higher among males than females, whereas there were no sex-related differences in seroprevalences of the other viral hepatitides (Table 3). Regarding ethnicity, the risk of HAV and HEV seropositivity was more than 4-fold higher in participants of Kyrgyz ethnicity than in Russian participants. In contrast, the risk of HCV seropositivity was two-times higher in Russian participants than in Kyrgyz participants (Table 3).

### 3.4. Notification Data

**Figure 2:**
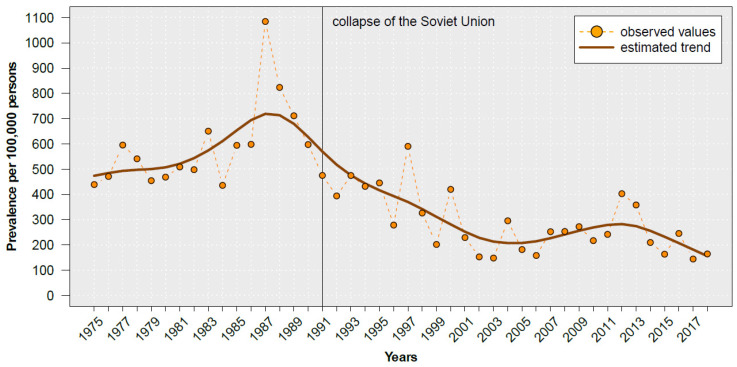
Prevalence of notified acute viral hepatitis in Kyrgyzstan, 1975–2018. Data source: Kyrgyz Ministry of Health [15]. The trend was fitted with a cubic smoothing spline.

The prevalence of notified acute viral hepatitis showed an increasing trend from 1975 to 1987, reaching the highest value of 1100 per 100,000 persons in 1987 (Figure 2).

**Figure 3:**
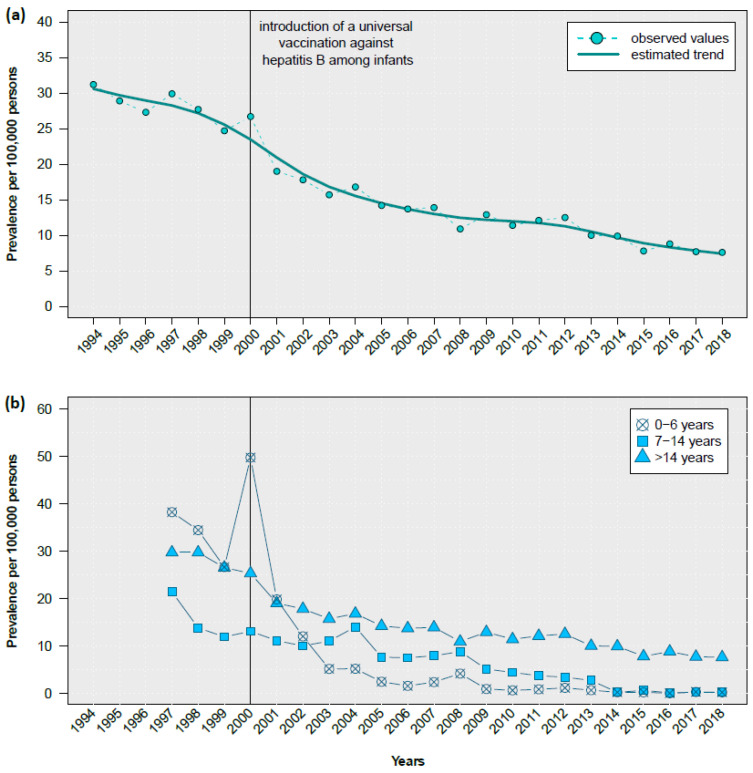
Prevalence of notified hepatitis B in total (**a**) and by age group (**b**) in Kyrgyzstan, 1994–2018. Data source: Kyrgyz Ministry of Health [15]. Upper panel: The trend was fitted with a cubic smoothing spline.

Since 1988, the prevalence has begun to decrease. Hepatitis B prevalence displayed a decreasing trend over the period of 1994 to 2018 (Figure 3a). The decrease was observed in all age groups, including children, adolescents, and adults, but was most prominent among 0- to 6-year-old children (Figure 3b).

## 4. Discussion

This is the first study to examine the seroprevalence of all viral hepatitides in a population-based study involving children and adults in a former Soviet Central Asian country, Kyrgyzstan. Very little is known about the seroepidemiology of viral hepatitides in this country. Our data suggest that the existing evidence is outdated, as the previous classification of endemicity patterns of viral hepatitides was primarily based on studies or reports from the early 1990s. The WHO observed in an assessment of viral hepatitis prevention and control in Kyrgyzstan that regular serological surveys are not conducted for any hepatitides in this country, including chronic hepatitis B and C [2].

*Hepatitis A.* Regarding HAV, Kyrgyzstan has previously been classified as a country with an intermediate endemicity [4]. The latter was defined as about half of the population infected with the virus by the age of 15 [4]. Our study confirms this endemic pattern. Namely, around half of boys under 10 years old were anti-HAV positive (and 40% of girls in the same age group). However, the proportion of seropositives increased in the age group of 30-to-39 years old, with nearly 90% seropositives. We are not aware of any national policy regarding hepatitis A vaccination. 

*Hepatitis B.* Kyrgyzstan is considered a highly endemic country for blood-borne viral hepatitis, including hepatitis B. Schweitzer et al. found that the only high-endemic country with regard to HBsAg in the WHO European Region with a prevalence of about 10% was Kyrgyzstan [5]. Karabaev et al. reported a much lower HBsAg seroprevalence in blood donors (3.6%; 3.4–3.8%) in Kyrgyzstan [7]. The HBsAg seroprevalence of 2.2% (1.5–3.3%) was even lower in the present study. We thus reclassified the country from a high (≥8%) to a lower endemicity country (2–4%). The decreasing prevalence trend has also been confirmed by the notification data and might be the result of several governmental programs to control and prevent HBV transmission, including the introduction of a universal hepatitis B vaccination among infants in the early 2000s in Kyrgyzstan. Since then, HBV vaccination coverage has reached 95% [16]. Of note, our data suggest that the introduction of universal HBV vaccination resulted in a shift of HBV burden to older ages: HBsAg seroprevalence was very low among children and adolescents and increased constantly after age 20. A very similar age pattern was observed in the notification data.

*Hepatitis C.* Botheju et al. conducted a systematic review and meta-analysis of HCV in Central Asia; HCV prevalence in Kyrgyzstan ranged in different populations, including blood donors, pregnant women, army recruits, and the general population, from 0.80% to 5.0% in studies conducted between 2004 and 2016, with a median HCV seroprevalence of 2.0% (95% CI: 1.7–2.4%) [9]. A seroprevalence of 2.5% (95% CI: 1.6–6.7%) among adults in Kyrgyzstan was reported in another systematic review [17]. Based on these and our results, Kyrgyzstan was classified as a country with high endemicity. In the present study, we observed a nearly two-fold higher seroprevalence of 3.8% (95% CI: 2.8–5.1%) than the two pooled estimates observed in the above-mentioned studies (i.e., 2% and 2.5% [9,17]). However, the seroprevalence estimates in more recent studies were similar or even higher than the estimate from our study (e.g., 2010 study, 5%; 2012, 4%; 2013, 2%; and 2014, 5%) [9]. In addition, the confidence intervals from the study by Gower et al. (95% CI: 1.6–6.7%) overlapped with the estimate from our study (3.8%), indicating no clear time trend. Follow-up assessments should be conducted to test whether this is a continuing trend.

*Hepatitis D.* Central Asian countries, including Kyrgyzstan, belong to countries with high HDV prevalence (20–40% of the HBV population) [18], which is close to the 15% detected in our study. 

*Hepatitis E.* All Central Asian countries are considered to be highly endemic countries with regard to HEV [19]. However, the epidemiologic situation in these countries, including Kyrgyzstan, is largely unknown. The WHO systematic review of global HEV prevalence did not find any single seroprevalence study in Azerbaijan, Georgia, or Kazakhstan [12]. Only a few studies were reported in Uzbekistan and Tajikistan. In Kyrgyzstan, HEV outbreaks were reported in the early 1990s, and Luhverchik et al. published a HEV seroprevalence of 4.8% (95% CI: 3.4–6.7%) [20], which agrees well with our estimate of 4.4% (95% CI: 3.3–5.8%). However, our nationally representative estimated prevalence was lower (3.3%). This relatively low HEV seroprevalence is similar to that usually reported from high-income countries, but prospective assessments should be conducted to assure that these values remain stable. Genotyping data for HEV may provide clues about the epidemiology of HEV; however, those data are essentially not available in Kyrgyzstan. We found only one study that reported HEV genotype 1 in human samples and genotype 3 isolated from a domestic pig [21]. 

*Ethnicity as a risk factor for seroprevalence of HAV, HBV, and HEV.* Hepatitis A and E viruses are predominantly water-borne viral pathogens that are transmitted via the fecal-oral route. Thus, low-income countries with poor hygienic conditions are highly endemic for HAV and HEV [4]. Kyrgyzstan is a lower middle-income country, with about one third of the population living below the poverty line [22]. We observed that Kyrgyz ethnicity was a risk factor for higher seroprevalence of these two viruses, whereas seroprevalence for the blood-borne pathogen HCV was preferentially associated with Russian ethnicity. The latter may be explained by a higher prevalence of risk factors for HCV, such as injecting drug use or alcohol abuse, among individuals of Russian ethnicity than Kyrgyz ethnicity. These results strongly suggest that ethnicity should be considered when devising educational and preventive measures for these pathogens in Kyrgyzstan. 

### Strengths and Limitations

The main strength of the study is that it provides the first population-based survey of the seroprevalence of all viral hepatitides among children and adults in Kyrgyzstan. However, it is limited in that the study population was not nationally representative in terms of selected sociodemographic data, including age and sex distribution. For example, the proportion of children in our study population was undersampled because the envisaged blood draw lowered willingness to participate in this age group. However, we applied post-stratification weighting to correct for demographic differences between the study and the source population and thus obtained nationally representative estimates. In addition, the study was conducted only in one region of the country, namely in the capital, Bishkek. Thus, it may not be representative of other regions of the country, in particular rural areas. Second, the sample size of the study was restricted due to available resources and thus underpowered to precisely detect HDV, which is a very low prevalent form of viral hepatitis. In addition, we were not able to conduct a multivariable risk factor analysis of seropositivity. Third, we used the same ELISA assays (and the same blood sample) to retest positive samples. This may not rule out a false-positive result in case of a false reactivity.

## 5. Conclusions

Our data provide the opportunity to reclassify Kyrgyzstan regarding the prevalence of viral hepatitides. Regarding hepatitis A and C, classification as a highly endemic country would now be warranted. Assuming that HBV and HDV seroprevalence is not substantially higher in areas outside the capital Bishkek (our study region), we suggest reclassifying the country from high to low intermediate endemicity of hepatitis B (i.e., seroprevalence 2–4% according to [5]) and to intermediate endemicity of hepatitis D (i.e., proportion of seropositives among HBV, 10–20% [18]). The observed anti-HEV seroprevalence resembles the seroepidemiology of high-income countries (i.e., a low endemicity pattern), but it remains to be clarified whether this is due to similar risk factors and modes of transmission.

## Figures and Tables

**Table 1:** Descriptive and health-related characteristics of the study population, Bishkek, Kyrgyzstan, 2018.

Characteristics	Study Population N = 1075		General Kyrgyz Population ^a^ N = 6,256,730	
	Number (*n*)	Percent (%)	Number (*n*)	Percent (%)
Sex				
Male	495	46.0	3,101,817	49.6
Female	580	54.0	3,154,913	50.4
Age groups				
<10 years	92	8.6	1,464,911	23.4
10–19 years	123	11.4	1,051,453	16.8
20–29 years	230	21.4	1,129,040	18.0
30–39 years	209	19.4	915,167	14.6
40–49 years	199	18.5	663,643	10.6
≥50 years	222	20.7	1,032,516	16.5
Ethnicity				
Kyrgyz	850	79.1	4,587,430	73.3
Russian	128	11.9	352,960	5.6
Uzbek	16	1.5	918,262	14.7
Kazakh	14	1.3	35,541	0.6
Ujgur	11	1.0	57,002	0.9
Other	7	0.7	305,535	4.9
Diabetes mellitus ^b^				
Yes	14	1.3	NA	NA
No	1045	97.2	NA	NA
Do not know	16	1.5	NA	NA
Myocardial infarction ^b^				
Yes	9	0.8	NA	NA
No	1057	98.3	NA	NA
Do not know	9	0.8	NA	NA
Asthma ^b^				
Yes	8	0.7	NA	NA
No	1057	98.3	NA	NA
Do not know	10	0.9	NA	NA
Cancer ^b^				
Yes	9	0.8	NA	NA
No	1055	98.1	NA	NA
Do not know	11	1.0	NA	NA
Rheumatoid arthritis ^b^				
Yes	34	3.2	NA	NA
No	1026	95.4	NA	NA
Do not know	15	1.4	NA	NA
Self-perceived health status ^b^				
Poor	51	4.7	NA	NA
Fair	122	11.3	NA	NA
Good	45	4.2	NA	NA
Very good	674	62.7	NA	NA
Excellent	183	17.0	NA	NA

^a^ The distribution of the general Kyrgyz population by sex and age is obtained from the National Statistical Committee of the Kyrgyz Republic (www.stat.kg accessed on 5 February 2023); ^b^ self-reported; NA—not available.

**Table 2:** Crude and weighted seroprevalence of viral hepatitis in a population-based sample, Bishkek, Kyrgyzstan.

Hepatitis	Unweighted Total Number of Study Participants ^a^	Number of Study Participants with Seropositive Results ^a^	Crude Proportion of Study Participants with Seropositive Results ^a^	Weighted Total Number of Study Participants ^b^	Weighted Proportion of Study Participants with Seropositive Results ^b^
	(N)	(*n*)	(% (95% CI))	(N)	(% (95% CI))
HAV	986	824	83.6 (81.1–85.8)	996	75.3 (72.5–77.9)
HBV	1074	33	3.1 (2.2–4.3)	1073	2.2 (1.5–3.3)
HCV	1074	51	4.7 (3.6–6.2)	1073	3.8 (2.8–5.1)
HDV	1004	6	0.60 (0.27–1.30)	1017	0.40 (0.15–1.01)
HEV	1065	47	4.4 (3.3–5.8)	1062	3.3 (2.4–4.5)

^a^ Unweighted number refers to the number of actually recruited study participants. ^b^ Poststratification weights were calculated with respect to the sex and age distribution of the general Kyrgyz population to obtain nationally representative estimates. The data were obtained from the National Statistical Committee of the Kyrgyz Republic (www.stat.kg, accessed on 5 February 2023). CI, confidence interval; HAV, hepatitis A virus; HBV, hepatitis B virus; HCV, hepatitis C virus; HDV, hepatitis D virus; HEV, hepatitis E virus.

**Table 3:** Sex-, age-, and ethnicity-adjusted odds ratios of seropositivity (results of four multivariable logistic regression models).

Variables	HAV AOR (95% CI)	HBV AOR (95% CI)	HCV AOR (95% CI)	HEV AOR (95% CI)
Sex				
male	reference	reference	reference	reference
female	1.08 (0.74–1.58)	0.88 (0.44–1.77)	**0.51 (0.29–0.93)**	0.66 (0.36–1.20)
Age (change per one year increase)				
	**1.08 (1.06–1.09)**	**1.03 (1.00–1.05)**	**1.04 (1.02–1.07)**	**1.05 (1.03–1.08)**
Ethnicity				
Kyrgyz	reference	reference	reference	reference
Russian	**0.21 (0.12–0.35)**	0.43 (0.10–1.84)	**2.39 (1.19–4.80)**	**0.22 (0.05–0.96)**
other	**0.37 (0.21–0.65)**	1.46 (0.50–4.29)	0.91 (0.27–3.05)	0.48 (0.11–2.03)

* Adjusted for all variables in the table. Values in bold indicate statistically significant results (*p* ≤ 0.05). AOR—adjusted odds ratio; CI—confidence interval; HAV—hepatitis A virus; HBV—hepatitis B virus; HCV—hepatitis C virus; HDV—hepatitis D virus; HEV—hepatitis E virus.

## Data Availability

The data presented in this study are available on request from the corresponding author.
